# Supplementary material for: MetaQTL: a package of new computational methods for the meta-analysis of QTL mapping experiments
Source: BMC Bioinformatics. 2007 Feb 8;8:49. doi: 10.1186/1471-2105-8-49 (PMC1808479; doi:10.1186/1471-2105-8-49)
Supplement: Additional File 7 — MetaQTL Package : jar file and tutorial. This Zip archive contains both the MetaQTL JAR file and the files of the tutorial. [file 1471-2105-8-49-S7.zip › org.inra.metaqtl/doc/org/metaqtl/algo/SVDAlgorithm.html]

SVDAlgorithm


|  |  |  |
| --- | --- | --- |
| **Overview** \| **Package** \| **Class** \| **Use** \| **Tree** \| **Deprecated** \| **Index** \| **Help** | |  |
| **PREV CLASS**   NEXT CLASS | **FRAMES**    **NO FRAMES**     **All Classes** |  |
| SUMMARY: NESTED \| FIELD \| CONSTR \| METHOD | DETAIL: FIELD \| CONSTR \| METHOD |  |


---


## org.metaqtl.algo Class SVDAlgorithm

```
java.lang.Object
  org.metaqtl.algo.SVDAlgorithm
```

---

``` public final class SVDAlgorithm extends java.lang.Object ```

This class defines methods to solve usual linear algebra
problems by using Singular Value Decomposition (SVD) strategy.
The methods of this class have been adapted from the
C implementation available in :
Numerical Recipes in C,
The Art of Scientific Computing by W. H. Press, B. P. Flannery,
S. A. Teukolsky and W. T. Vetterling.
1990, Cambridge University Press, Cambridge, England
ISBN 0-521-35465-X (the book)

---

**Constructor Summary**

`SVDAlgorithm()`


| **Method Summary** | |
| --- | --- |
| `static double[]` | `SVDBackSub(double[][] U, double[] w, double[][] V, double[] b, int n, int m)`             Solves A.X=B for a vector X, where `A` is specified by the matrix `U`,`V` and the vector `w` as returned by the SVDecomposition() method. |
| `static void` | `SVDecomposition(double[][] A, double[][] V, double[] w, int m, int n)`             Given a matrix `A`, this routine computes its singular value decomposition A = U.W.V' . |
| `static MultiFitResult` | `SVDMFit(double[][] X, double[] y, double[] w, int m, int n)`             Given a set of data points y[1..m] with indiviudal weights w[1..m], use chi2 minimization to determine the coefficients a[1..n] of the fitting function y = X\*a. |

**Methods inherited from class java.lang.Object**

`clone, equals, finalize, getClass, hashCode, notify, notifyAll, toString, wait, wait, wait`

**Constructor Detail**

### SVDAlgorithm

```
public SVDAlgorithm()
```


**Method Detail**

### SVDMFit

```
public static MultiFitResult SVDMFit(double[][] X,
                                     double[] y,
                                     double[] w,
                                     int m,
                                     int n)
```

:   Given a set of data points y[1..m] with indiviudal weights
    w[1..m], use chi2 minimization to determine the coefficients a[1..n] of the fitting
    function y = X\*a. Here we solve the fitting equations using singular value
    decomposition of the X matrix.

    :   **Parameters:**: `X` - the design matrix.: `y` - the observations.: `w` - the weigths, i.e the invsers of the standard deviations.: `m` - the number of observations.: `n` - the number of parameters. **Returns:**: the parameter estimates. **See Also:**: `MultiFitResult`, `SVDBackSub(double[][], double[], double[][], double[], int, int)`, `SVDecomposition(double[][], double[][], double[], int, int)`

---


### SVDBackSub

```
public static double[] SVDBackSub(double[][] U,
                                  double[] w,
                                  double[][] V,
                                  double[] b,
                                  int n,
                                  int m)
```

:   Solves A.X=B for a vector X, where `A` is specified by the
    matrix `U`,`V` and the vector `w`
    as returned by the SVDecomposition() method. No input are destroyed, so
    the method may be called sequentially with different `b` 's.
    Note that this routine presumes that you have already zeroed the small
    wj's. It does not do this for you. If you haven't zeroed the small wj's,
    the this routine is just as ill-conditioned as any direct method, and you
    are misusing SVD.

    :   **Parameters:**: `A` - the design matrix.: `w` - the singular values.: `V` - the right singular vectors.: `b` - the vector. **Returns:**: X the vector solution of A.X = b.

---


### SVDecomposition

```
public static void SVDecomposition(double[][] A,
                                   double[][] V,
                                   double[] w,
                                   int m,
                                   int n)
```

:   Given a matrix `A`, this routine computes its singular
    value decomposition A = U.W.V' . The matrix U replaces A on output.
    The diagonal matrix of singular values W is output as a vector
    `w`. The matrix V (not the transpose V') is output as
    the matrix `V`. The original routine comes from :
    Forsythe, G.E., Malcolm, M.A. and Moler, C.B. 1977, Computer Methods
    for Mathematical Computations (Englewood Cliffs, NJ: Prentice-Hall),
    Chapter 9. which is in turned based on the routine from : Golub, G.H.
    and Van Loan, C.F. 1989, Matrix Computations, 2nd ed. (Baltimore:
    Johns Hopkins University Press), p8.3 and chapter 12.

    :   **Parameters:**: `A` - the original matrix, after the left singular vectors.: `V` - the right singular vectors.: `w` - the singular values.


---


|  |  |  |
| --- | --- | --- |
| **Overview** \| **Package** \| **Class** \| **Use** \| **Tree** \| **Deprecated** \| **Index** \| **Help** | |  |
| **PREV CLASS**   NEXT CLASS | **FRAMES**    **NO FRAMES**     **All Classes** |  |
| SUMMARY: NESTED \| FIELD \| CONSTR \| METHOD | DETAIL: FIELD \| CONSTR \| METHOD |  |


---
